# Fresh Frozen Plasma in Cases of Acute Upper Gastrointestinal Bleeding Does Not Improve Outcomes

**DOI:** 10.3389/fmed.2022.934024

**Published:** 2022-07-14

**Authors:** Shuang Liu, Xiaoming Zhang, Joseph Harold Walline, Xuezhong Yu, Huadong Zhu

**Affiliations:** ^1^Emergency Department, State Key Laboratory of Complex Severe and Rare Diseases, Peking Union Medical College Hospital, Chinese Academy of Medical Science and Peking Union Medical College, Beijing, China; ^2^Department of Nursing, Peking Union Medical College Hospital, Beijing, China; ^3^Accident and Emergency Medicine Academic Unit, Prince of Wales Hospital, The Chinese University of Hong Kong, Hong Kong, China

**Keywords:** acute upper gastrointestinal bleeding, fresh frozen plasma, mortality, rebleeding, blood transfusion

## Abstract

**Background:**

Blood products are commonly transfused in patients with acute upper gastrointestinal bleeding (UGIB). There exists considerable practice variation and less evidence to guide fresh frozen plasma transfusion in patients with UGIB. The aim of this study was to explore any association between fresh frozen plasma transfusion following acute UGIB and clinical outcomes.

**Methods:**

This was a prospective, observational, multicenter study conducted at 20 tertiary hospitals in China. Patients with acute UGIB with an international normalized ratio ≤ 2.0 at emergency department admission were included. Multivariate logistic regression models were used to examine and quantify any clinical associations.

**Results:**

A total of 976 patients (61.57 ± 15.79 years old, 73.05% male) were included, of whom 17.42% received fresh frozen plasma transfusion. The overall 90-day mortality and rebleeding rates were 10.20 and 12.19%, respectively. After adjusting for confounding factors, transfusion of fresh frozen plasma during hospitalization was associated with higher 90-day mortality [odd ratio (OR), 2.36; 95% confidence interval (CI), 1.36–4.09; *p* = 0.002] but not rebleeding (OR, 1.5; 95% CI; 0.94-2.54; *p* = 0.085). In a subgroup analysis, patients with an international normalized ratio <1.5 who were treated with fresh frozen plasma were associated with both significantly higher 90-day mortality (OR, 2.78; 95% CI, 1.49–5.21; *p* = 0.001) and rebleeding (OR, 2.02; 95% CI, 1.16–3.52; *p* = 0.013), whereas in patients with an international normalized ratio between 1.5 and 2, we did not find any significant correlation.

**Conclusion:**

This study found an association between fresh frozen plasma transfusion following acute UGIB and elevated 90-day mortality. Both 90-day mortality and rebleeding risk were significantly higher in patients with an international normalized ratio < 1.5. Fresh frozen plasma transfusion in acute UGIB does not improve the poor outcomes (Chinese Clinical Trial registry, Number ChiCTR1900028676).

## Introduction

Acute upper gastrointestinal bleeding (UGIB) is a relatively common medical emergency associated with significant morbidity and mortality worldwide, with an incidence of 80–150 per 100,000 people each year (1–4). Despite recent advancements in pharmaceuticals and endoscopic hemostasis, the 30-day mortality from UGIB remains 5–14% (1–5).

Transfusion of blood components is integral to the management of patients with acute UGIB, but blood transfusion practice for acute UGIB is mainly empirical, and the evidence base of crystal-restricted empirical blood transfusion algorithms mainly comes from the study of major bleeding in trauma (6–13). Practice guidelines about UGIB recommend urgent reversal in all patients presenting with serious, life-threatening bleeding (i.e., hemodynamic instability or shock), either in the case of therapeutic or supratherapeutic international normalized ratio (INR) elevations (14). While the early benefits of fresh frozen plasma (FFP) infusion for patients with severe gastrointestinal bleeding are self-evident, its long-term effects on patients with severe UGIB and the benefits for less severe cases of UGIB remain unclear (6).

In most patients with UGIB, FFP infusions are not only used as part of resuscitation, but also are used to prevent or treat bleeding, usually guided by laboratory coagulation parameters (6). The causes of gastrointestinal hemorrhage and coagulopathy in patients with UGIB are different from those in trauma. In the case of gastrointestinal bleeding, the most common causes of coagulopathy are hepatic cirrhosis and oral anticoagulants (15, 16). However, prolonged INR in the setting of cirrhosis is not thought to be predictive of bleeding risk, so it is not clear what is the role of FFP in the setting of very mild prolongation of INR in cirrhotic patients (17, 18). In the case of coagulopathy caused by oral anticoagulant medication, prothrombin complex concentrate is most appropriate for reversing the effects of vitamin K antagonists. (6) The evidence for the use of blood components to reverse the anticoagulant effects of oral anticoagulants is poor.

In the absence of high-quality data, we performed a large prospective, real-world, observational study to determine the relationship between an FFP infusion within 72 h following presentation to an emergency department (ED) in patients with UGIB with an INR ≤2 at admission and key clinical outcomes (90-day mortality and rebleeding).

## Materials and Methods

### Study Design and Participants

This was a prospective, multicenter, non-interventional, real-world study (acute upper gastrointestinal real-world research, AUGUR) in China. The study sample included all non-trauma adult (age ≥18 years) patients diagnosed with UGIB who were admitted to an acute care hospital *via* the ED between 30 June 2020 and 10 February 2021. The diagnosis of UGIB was based on the presence of hematemesis, coffee ground emesis, and melena. Patients who refused to sign the participation consent form were excluded. In this study, to make the results more clinically meaningful, we excluded patients with an INR >2 at the time of admission. This study was conducted at 20 tertiary hospitals from 20 (out of an invited 31) different provinces, autonomous regions, or independent municipalities and was approved by the Institutional Ethics Review Boards of all 20 hospitals. Informed consent was obtained from all enrolled patients.

### Definition of Outcomes

The primary outcome was the association between FFP transfusion and 90-day mortality. The secondary outcome was the association between FFP transfusion and rebleeding. Such outcomes were monitored from admission to the hospital or the onset of bleeding at admission to the hospital (for in-hospital patients) for up to 90 days. Mortality was defined as death from any cause within 90 days following the initial presentation. Rebleeding was defined by a recurrent episode of hematemesis, coffee ground emesis, melena, or both, with either shock or a decrease in hemoglobin concentration of at least 2 g/dl 24 h following the initial treatment and stabilization. All data were recorded for the full duration of the initial medical encounter.

### Covariates

Recorded variables included age, gender, vital signs at triage, comorbidities, relevant past medical history, any concomitant intake of medications in 6 months preceding the bleeding episode, physical examination findings and laboratory data (hemodynamic data, complete blood count, and coagulation profile results), and any resuscitative measures employed. Endoscopic reports included the identification of any bleeding lesions or stigmata of recent hemorrhage. Both surgical and angiographic therapies, as well as any pharmacologic therapies administered for hemorrhage were recorded. Thrombotic in hospital refers to the occurrence of myocardial infarction, cerebral infarction, pulmonary embolism, or lower extremity thrombosis after admission.

### Statistical Analysis

For baseline characteristics, variables are reported as mean ± standard deviation (s.d.) or proportions when they were continuous data or categorical data, respectively. The comparisons between different groups were performed using unpaired *t*-tests, chi-square, Fisher's exact, or Wilcoxon rank testing, as appropriate. Multiple logistic regression models were created to analyze possible independent relationships between FFP transfusion and mortality and rebleeding. We also listed different adjusted models as follows: unadjusted model 1; model 2: adjusted for age and gender; model 3 for fully adjustment (age, gender, systolic blood pressure, cirrhosis, hemoglobin at admission, hematemesis, peptic ulcer bleeding, thrombotic in hospital, red blood cells transfusion). Briefly, the potential confounding factors were based on clinical considerations that related to baseline patient characteristics or therapies administered and also both associated with FFP transfusion and clinical outcomes (90-day mortality and rebleeding). Independent variables were tested for multicollinearity and only included in the model if absent. Results are presented as odds ratios (OR) with 95% confidence intervals (95% CIs). *P*-values < 0.05 were considered statistically significant. Statistical analyses were performed with the R statistical software packages (The R Foundation, Vienna, Austria) and Empowerstats (Solutions, Inc., Boston, MA, USA).

## Results

### Patient Characteristics

Of the initial sample, 60 patients were excluded due to lower gastrointestinal bleeding, 56 patients were excluded due to unavailable laboratory data, 12 patients were excluded due to loss to follow-up, and 96 patients were excluded due to an INR >2 at admission. Finally, a total of 976 patients with UGIB from 20 hospitals in 20 (out of a surveyed 31) provinces, autonomous regions, or independent municipalities were enrolled in this analysis. During the study period, all patients enrolled presented first to the ED (i.e., there were no enrolments of already admitted patients) and all were identified as having an UGIB. The overall mean age was 61.57 ± 15.79 years, 24.39% of patients had liver cirrhosis, and 5.12% of patients were taking oral anticoagulants. Among all patients, 76.43% of patients completed an endoscopic examination, and 17.42% of patients received FFP transfusion within 72 h after ED admission. Out of all the patients, 10.20% of died and 12.19% of experienced rebleeding. The characteristics of all patients, including those who received FFP transfusion within 72 h of admission and those who did not, are summarized in Table 1.

**Table 1:** Baseline characteristics of the two groups.

**Characteristics**	**All patients**	**No FFP**	**FFP**	**Standard diff**.	***P*** -value^*^
	***N*** **= 976**	***N*** **= 806**	***N*** **= 170**		
	Mean ± SD^*^/*N* (%)			(95% CI)	
Age (yr)	61.57 ± 15.79	61.74 ± 15.92	60.75 ± 15.19	0.06 (−0.10, 0.23)	0.238
Male sex	713 (73.05%)	587 (72.83%)	126 (74.12%)	0.03 (−0.14, 0.19)	0.731
**Vital signs**					
SBP (mmHg)^*^	116.62 ± 23.72	117.83 ± 23.11	110.90 ± 25.73	0.28 (0.12, 0.45)	<0.001
HR (bpm)^*^	92.28 ± 19.51	91.62 ± 19.26	95.42 ± 20.41	0.19 (0.03, 0.36)	0.021
GCS	14.86 ± 0.74	14.87 ± 0.73	14.83 ± 0.78	0.05 (−0.11, 0.22)	0.341
**Laboratory (on admission)**					
HGB (g/dL)^*^	9.02 ± 2.93	9.27 ± 2.97	7.82 ± 2.43	0.54 (0.37, 0.70)	<0.001
PLT (× 10^9^/*L*)^*^	191.93 ± 96.32	199.31 ± 94.09	156.93 ± 99.32	0.44 (0.27, 0.60)	<0.001
ALB (g/L)^*^	34.36 ± 8.02	35.21 ± 7.82	30.07 ± 7.63	0.67 (0.49, 0.84)	<0.001
BUN (mmol/L)^*^	11.76 ± 8.05	11.69 ± 7.77	12.07 ± 9.28	0.04 (−0.12, 0.21)	0.578
INR^*^	1.20 ± 0.24	1.17 ± 0.21	1.35 ± 0.28	0.74 (0.57, 0.91)	<0.001
APTT (s)^*^	30.67 ± 6.30	30.36 ± 5.87	32.08 ± 7.88	0.25 (0.08, 0.41)	0.001
**Medications on presentation**					
PPI	84 (8.61%)	64 (7.94%)	20 (11.76%)	0.13 (−0.04, 0.29)	0.106
Aspirin	136 (13.93%)	121 (15.01%)	15 (8.82%)	0.19 (0.03, 0.36)	0.034
NSAIDs^*^	79 (8.09%)	70 (8.68%)	9 (5.29%)	0.13 (−0.03, 0.30)	0.141
Anticoagulant drugs	20 (2.05%)	15 (1.86%)	5 (2.94%)	0.07 (−0.10, 0.24)	0.368
**Comorbidities**					
Coronary heart disease	140 (14.34%)	122 (15.14%)	18 (10.59%)	0.14 (−0.03, 0.30)	0.124
Hypertension	312 (31.97%)	271 (33.62%)	41 (24.12%)	0.21 (0.05, 0.38)	0.016
Atrial fibrillation	20 (2.05%)	15 (1.86%)	5 (2.94%)	0.07 (−0.09, 0.24)	0.366
Diabetes mellitus	180 (18.44%)	153 (18.98%)	27 (15.88%)	0.08 (−0.08, 0.25)	0.344
Cirrhosis	238 (24.39%)	161 (19.98%)	77 (45.29%)	0.56 (0.39, 0.73)	<0.001
Chronic kidney disease	42 (4.30%)	36 (4.47%)	6 (3.53%)	0.05 (−0.12, 0.21)	0.584
Gastrointestinal tumors	50 (5.12%)	39 (4.84%)	11 (6.47%)	0.07 (−0.09, 0.24)	0.380
**Onset symptoms**					
Hematemesis	514 (52.66%)	398 (49.38%)	116 (68.24%)	0.39 (0.22, 0.56)	<0.001
Melena	752 (77.05%)	626 (77.67%)	126 (74.12%)	0.08 (−0.08, 0.25)	0.317
**Accompanying symptoms**					
Confusion	29 (2.97%)	20 (2.48%)	9 (5.29%)	0.15 (−0.02, 0.31)	0.050
Syncope	81 (8.30%)	67 (8.31%)	14 (8.24%)	0.00 (−0.16, 0.17)	0.973
Endoscopy		612 (75.93%)	134 (78.82%)	0.07 (−0.10, 0.23)	0.419
First Endoscopy time				0.16 (−0.01, 0.32)	0.462
<12 h	277 (28.38%)	235 (29.16%)	42 (24.71%)		
12–24 h	173 (17.73%)	143 (17.74%)	30 (17.65%)		
24–48 h	114 (11.68%)	90 (11.17%)	24 (14.12%)		
≥48 h	180 (18.44%)	143 (17.74%)	37 (21.76%)		
**Endoscopy finding**					
No abnormalities seen	5 (0.51%)	4 (0.50%)	1 (0.59%)	0.01 (−0.15, 0.18)	0.879
Variceal bleeding	179 (18.34%)	106 (13.15%)	73 (42.94%)	0.70 (0.53, 0.87)	<0.001
Peptic ulcer bleeding	390 (39.96%)	353 (43.80%)	37 (21.76%)	0.48 (0.32, 0.65)	<0.001
Esophagitis/gastritis/duodenitis	80 (8.20%)	70 (8.68%)	10 (5.88%)	0.11 (−0.06, 0.27)	0.226
Dieulafoy's lesion	4 (0.41%)	2 (0.25%)	2 (1.18%)	0.11 (−0.05, 0.28)	0.085
Mallory-weiss syndrome	32 (3.28%)	31 (3.85%)	1 (0.59%)	0.22 (0.06, 0.39)	0.030
Upper gastrointestinal tumors	8 (0.82%)	7 (0.87%)	1 (0.59%)	0.03 (−0.13, 0.20)	0.713
Other findings	11 (1.13%)	10 (1.24%)	1 (0.59%)	0.07 (−0.10, 0.23)	0.464
**Treatment after admission**					
Interventional radiology	33 (3.38%)	19 (2.36%)	14 (8.24%)	0.26 (0.10, 0.43)	<0.001
Endoscope treatment	294 (30.12%)	214 (26.55%)	80 (47.06%)	0.44 (0.27, 0.60)	<0.001
RBC transfusion^*^	439 (44.98%)	294 (36.48%)	145 (85.29%)	1.16 (0.98, 1.33)	<0.001
PLT transfusion	19 (1.95%)	9 (1.12%)	10 (5.88%)	0.26 (0.10, 0.43)	<0.001
Emergency surgery	26 (2.66%)	17 (2.11%)	9 (5.29%)	0.17 (0.00, 0.33)	0.019
**Complications in the hospital**					
Thrombotic event in hospital	42 (4.30%)	30 (3.72%)	12 (7.06%)	0.15 (−0.02, 0.31)	0.051
Pulmonary edema at 48 h	10 (1.02%)	6 (0.74%)	4 (2.35%)	0.13 (−0.03, 0.30)	0.058

*SD, standard deviation; SBP, systolic blood pressure; HR, heart rate; GCS, Glasgow coma scale; HGB, hemoglobin; PLT, Platelet; ALB, albumin; BUN, blood urea nitrogen; INR, international normalized ratio; APTT, activated partial thromboplastin time; PPI, proton pump inhibitor; NSAIDs, non-steroidal anti-inflammatory drugs; RBC: red blood cells*.

* *Chi-square test for association and non-parametric Wilcoxon for continuous variables*.

### Comparison Between Patients Who Received FFP Transfusion and Those Without FFP Transfusion

Among the 170 patients who received FFP transfusion, 45.29% had liver cirrhosis, and the proportion of patients was significantly higher than that of the group who did not receive FFP transfusion. In addition, patients who received FFP transfusion were more likely to have hematemesis, and their admission systolic blood pressure, hemoglobin, and albumin average were lower than those who did not receive FFP transfusion. Overall, more patients in the FFP blood transfusion group showed signs of initial hemodynamic instability upon admission. Endoscopic results showed that the proportion of variceal bleeding was higher in the FFP transfusion group, while the proportion of peptic ulcers was higher in the non-plasma transfusion group. In examining treatments after admission, the proportion of the participants in the FFP transfusion group who received red blood cell transfusions, endoscopic treatments, or emergency surgery was higher than those in the non-FFP transfusion group (Table 1).

### The Association Between Transfusion and Mortality or Rebleeding

All-cause 90-day mortality and rebleeding were higher in FFP transfused vs. non-transfused patients (mortality: 20.00 vs. 8.19%, *p* < 0.001; rebleeding: 21.18 vs. 10.30%, *p* < 0.001) on univariate analysis. Other characteristics significantly associated with death and rebleeding in univariate analysis included age, liver cirrhosis, gastrointestinal tumors, hematemesis, systolic blood pressure (SBP), hemoglobin (HGB), albumin (ALB), peptic ulcer bleeding, and thrombotic events in hospital (Table 2). On multivariate analysis after adjusting for confounding variables, including age, gender, SBP, HGB, liver cirrhosis, hypertension, confusion, hematemesis, peptic ulcer bleeding, thrombosis in hospital, and RBC transfusion, an FFP transfusion was an independent predictor of 90-day mortality (OR, 2.36; 95% CI, 1.36–4.09; *p* = 0.002) but not for rebleeding (OR, 1.55; 95% CI, 0.94–2.54; *p* = 0.085) (Table 3).

**Table 2:** Univariate analysis of 90-day mortality and rebleeding.

**Factor**	**90-day mortality**		**Rebleeding**	
	**OR (95% CI)**	***P*** **-value**	**OR (95% CI)**	***P*** **-value**
Age (yr)	1.03 (1.02, 1.05)	<0.0001	1.02 (1.01, 1.03)	0.005
Male sex	0.94 (0.59, 1.50)	0.802	0.79 (0.52, 1.20)	0.278
**Medicine on presentation**				
PPI	1.20 (0.60, 2.41)	0.600	1.22 (0.64, 2.33)	0.540
Aspirin	0.91 (0.50, 1.69)	0.776	0.88 (0.49, 1.56)	0.655
NSAIDs	1.14 (0.55, 2.36)	0.726	1.05 (0.52, 2.09)	0.895
Anticoagulant drugs	1.56 (0.45, 5.42)	0.483	0.37 (0.05, 2.81)	0.339
**Comorbidity**				
Coronary heart disease	1.25 (0.72, 2.19)	0.425	1.16 (0.68, 1.96)	0.590
Hypertension	1.35 (0.88, 2.07)	0.173	1.00 (0.66, 1.51)	0.993
Atrial fibrillation	0.97 (0.22, 4.25)	0.971	0.80 (0.18, 3.48)	0.763
Diabetes mellitus	1.20 (0.72, 2.00)	0.487	1.07 (0.66, 1.74)	0.791
Cirrhosis	1.61 (1.03, 2.52)	0.036	2.58 (1.73, 3.84)	<0.0001
Chronic kidney disease	0.43 (0.10, 1.79)	0.245	0.54 (0.16, 1.78)	0.314
Gastrointestinal tumors	4.72 (2.50, 8.90)	<0.0001	2.42 (1.23, 4.77)	0.011
**Onset symptoms**				
Hematesis	1.77 (1.15, 2.72)	0.010	1.62 (1.09, 2.41)	0.016
Melaena	0.49 (0.31, 0.75)	0.001	0.75 (0.49, 1.15)	0.187
**Accompanying symptoms**				
Confusion	3.54 (1.52, 8.22)	0.003	1.16 (0.40, 3.39)	0.789
Syncope	0.96 (0.45, 2.05)	0.909	0.77 (0.36, 1.65)	0.507
**Vital signs**				
SBP (mmHg)	0.98 (0.98, 0.99)	0.001	1.00 (0.99, 1.01)	0.824
HR (bpm)	1.01 (1.00, 1.02)	0.016	1.01 (1.00, 1.02)	0.273
GCS	0.57 (0.45, 0.72)	<0.0001	0.96 (0.76, 1.21)	0.714
**Laboratory (on admission)**				
HGB (g/dL)	0.99 (0.98, 1.00)	0.005	0.99 (0.98, 0.99)	0.000
PLT (× 10^9^/*L*)	1.00 (1.00, 1.00)	0.695	1.00 (0.99, 1.00)	0.005
ALB (g/L)	0.91 (0.88, 0.95)	<0.0001	0.95 (0.92, 0.97)	0.000
BUN (mmol/L)	1.01 (0.99, 1.03)	0.417	1.00 (0.97, 1.02)	0.741
APTT (s)	1.06 (1.03, 1.09)	0.000	1.05 (1.02, 1.08)	0.001
Endoscopy	0.36 (0.23, 0.55)	<0.0001	0.71 (0.46, 1.08)	0.110
No abnormalities seen	0.00 (0.00, Inf)	0.984	11.06 (1.83, 66.87)	0.009
Variceal bleeding	1.38 (0.84, 2.27)	0.205	2.38 (1.56, 3.65)	<0.0001
Peptic ulcer bleeding	0.28 (0.16, 0.47)	<0.0001	0.38 (0.24, 0.60)	<0.0001
Esophagitis/gastritis/duodenitis	0.44 (0.16, 1.23)	0.116	0.26 (0.08, 0.84)	0.025
Dieulafoy's lesion	0.00 (0.00, Inf)	0.985	0.00 (0.00, Inf)	0.977
Mallory-weiss syndrome	0.28 (0.04, 2.03)	0.206	0.74 (0.22, 2.46)	0.622
Upper gastrointestinal tumors	1.25 (0.15, 10.30)	0.833	1.03 (0.13, 8.44)	0.979
Other finding	0.87 (0.11, 6.90)	0.899	0.72 (0.09, 5.66)	0.753
**Treatment after admission**				
Interventional radiography	1.59 (0.60, 4.23)	0.349	2.40 (1.06, 5.45)	0.037
Endoscope treatment	0.63 (0.38, 1.03)	0.064	1.10 (0.73, 1.66)	0.646
RBC transfusion	1.50 (0.99, 2.27)	0.057	1.97 (1.33, 2.90)	0.001
FFP transfusion	2.80 (1.78, 4.41)	<0.0001	2.34 (1.52, 3.61)	0.000
PLT transfusion	3.24 (1.14, 9.19)	0.027	3.45 (1.28, 9.25)	0.014
Emergency surgery	0.72 (0.17, 3.11)	0.665	1.32 (0.45, 3.90)	0.615
**Complications in the hospital**				
Thrombotic event in hospital	3.85 (1.90, 7.78)	0.000	2.71 (1.33, 5.56)	0.006
Pulmonary edema in 48 h	6.04 (1.68, 21.79)	0.006	1.81 (0.38, 8.65)	0.455

*SD, standard deviation; SBP, systolic blood pressure; HR, heart rate; GCS, Glasgow coma scale; HGB, hemoglobin; PLT, Platelet; ALB, albumin; BUN, blood urea nitrogen; INR, international normalized ratio; APTT, activated partial thromboplastin time; PPI, proton pump inhibitor; NSAIDs, non-steroidal anti-inflammatory drugs; RBC: red blood cells*.

**Table 3:** Association between FFP transfusion and 90-day mortality or rebleeding by multiple regression.

**Exposure**	**90-day mortality**			**Rebleeding**		
	**OR (95% CI)** ***P*****-value**			**OR (95% CI)** ***P*****-value**		
	**Non-adjusted**	**Adjust I**	**Adjust II**	**Non-adjusted**	**Adjust I**	**Adjust II**
No transfusion	1	1	1	1	1	1
Transfusion	2.80 (1.78, 4.41) <0.0001	3.03 (1.91, 4.82) <0.0001	2.36 (1.36, 4.09) 0.002	2.34 (1.52, 3.61) 0.000	2.41 (1.56, 3.73) <0.0001	1.55 (0.94, 2.54) 0.085

*Non-adjusted model adjusted for None. Adjust I model adjusted for Age; Gender. Adjust II model adjusted for Age; Gender; SBP; HGB; cirrhosis; hypertension; confusion; hematesis; peptic ulcer bleeding; thrombotic event in hospital; RBC transfusion*.

### Subgroup Analysis for the Association Between FFP Transfusion and Mortality or Rebleeding Based on INR

We then performed a subgroup analysis by dividing all patients into an INR <1.5 group and the INR 1.5–2 group, according to the admission data. After multivariate adjustment, we found that FFP transfusion in the INR <1.5 group was associated with a higher 90-day mortality rate (OR, 2.78; 95% CI, 1.49–5.21; *p* = 0.001) and rebleeding rate (OR, 2.02; 95% CI, 1.16–3.52; *p* = 0.013). There was no significant correlation between FFP transfusion and 90-day mortality or rebleeding in patients with an INR between 1.5 and 2 (Table 4).

**Table 4:** Association between FFP transfusion of 90-day mortality or rebleeding in subgroup analysis.

**Variable**	***N***	**90-day mortality**		**Rebleeding**	
		**OR (95% CI)**	***P*** **-value**	**OR (95% CI)**	***P*** **-value**
INR <1.5	862	2.78 (1.49, 5.21)	0.001	2.02 (1.16, 3.52)	0.013
1.5 ≤ INR ≤ 2	114	0.64(0.17, 2.38)	0.502	0.49 (0.14, 1.71)	0.263

*Adjusted for Age; Gender; SBP; HGB; liver cirrhosis; hypertension; confusion; hematesis; peptic ulcer bleeding; thrombotic event in hospital; RBC transfusion*.

## Discussion

This large, prospective, observational, real-world study examined the association between FFP transfusions in patients with acute UGIB and the clinical outcomes of mortality and rebleeding. We found that any transfusion of FFP within 72 h of admission was associated with an increased risk of all-cause 90-day mortality. We also observed a trend toward increased rebleeding, which fell short of statistical significance, perhaps because of a lack of statistical power. Unlike the 30-day mortality or in-hospital mortality selected by most previous studies, we chose the 90-day mortality and rebleeding rate as our outcome indicators to avoid missing information on patients with adverse events after discharge. The sample size was relatively large with nearly 1,000 patients with UGIB included, which allowed us to examine the association of an FFP transfusion with the outcomes of patients with acute UGIB.

To ensure hemodynamic stability, many patients with acute gastrointestinal bleeding will receive high-density resuscitation [such as fluid resuscitation, FFP, and RBC transfusion(s)] within a short period of time after arrival in ED. In recent decades, there has been great progress in the exploration of fluid resuscitation and red blood cell infusion strategies, but so far, published data on the use of FFP in patients with UGIB are still limited and inconclusive (14, 17). UK NICE guidance on UGIB recommends transfusion of frozen plasma in actively bleeding patients if the INR is more than 1.5 (16). Australian guidelines also recommend that FFP transfusion in the setting of major RBC transfusion be guided by both the coagulation profile and the clinical scenario, with an INR of more than 1.5 likely reflecting a coagulopathy requiring correction by FFP and/or other coagulation factors (18). In our study, a total of 179 patients with UGIB received FFP transfusion within 72 h of admission. There are 67 patients admitted to the hospital with an INR between 1.5 and 2 who did not receive plasma transfusion. Among all 976 patients admitted to the hospital with an INR ≤2, 104 patients were admitted to the hospital with an SBP <90 mmHg, but only 29 of them underwent a plasma infusion. This captures the wide variability in medical management for patients with acute UGIB, indicating clinical uncertainty regarding optimal practice.

Coagulopathic patients with critical UGIB are a focus of ongoing clinical research. In most patients with UGIB without a coagulopathy, there are few studies on whether a FFP transfusion will cause harm or be ineffective to patients (19, 20). Hence, we only included patients with UGIB with an INR ≤2.0, as this population of patients would probably not benefit from FFP. In a multicenter study of non-variceal UGIB, there was an association between FFP with greater mortality (30-day and 1-year survival outcomes). These poor outcomes were independent of RBC transfusion and were dependent on the FFP dose (19). One small cohort study using a historical comparison group showed that aggressive volume resuscitation, including correction of coagulopathy (target = INR <1.8), led to an improved mortality (20).

In addition, the value of FFP on survival outcomes has also been questioned outside of UGIB cases in the trauma, intensive care, and cardiac surgery literature (21–23). Reported adverse effects of FFP transfusion include transfusion-associated lung injury and circulatory overload, with the risk of acute lung injury being higher in patients who received FFP and PLTs than in those who received only RBCs (24).

In our study, the group of patients receiving FFP was much sicker (lower albumin level, lower blood pressure, more hemodynamic instability, more variceal bleeding). In these cases, bleeding may not be due to anticoagulation reasons, and transfusion with clotting factors cannot prevent or treat their underlying UGIB. Indeed, after adjusting for confounding factors, FFP did not reduce patient deaths, and, in fact, the FFP group had a higher 90-day mortality rate (but not a higher rebleeding risk). Among the adverse events recorded in our study, we also observed a correlation between plasma infusion and a trend of increased rates of pulmonary edema within 48 h and overall thrombotic events in the hospital, but these trends were not statistically significant, which may be due to a relatively small number of adverse events in our sample.

As in most studies, INR ≥1.5 is used as the criterion for judging coagulopathy, and coagulopathy is independently related to the death of patients with UGIB (16), we conducted a subgroup analysis of patients (admission INR <1.5 or INR between 1.5 and 2.0), and found that in patients with INR <1.5, transfusion of FFP within 72 h not only increased the 90-day mortality, but also increased the risk of rebleeding, but this was not found in the 1.5–2.0 INR group. This also indicates that there is a correlation between bleeding and anticoagulation in patients with INR 1.5–2, and FFP transfusion can prevent or treat their potential bleeding. Therefore, we speculate that in patients who have not developed coagulopathy, a “less is more” philosophy may be better.

Although it may seem counterintuitive to question the value of an FFP transfusion in UGIB, numerous studies in other disciplines that have assessed critically ill patient populations have suggested associations between FFP transfusions and adverse patient outcomes, including death, which persist after appropriate adjustment. In patients with UGIB, a restrictive blood transfusion strategy may be expanded to restrictive blood component transfusion strategy (red blood cells and plasma). Different from coagulopathy caused by trauma, most patients with UGIB have a coagulopathy associated with either oral anticoagulants or liver disease (7, 25). In the case of coagulopathy caused by oral anticoagulant medications, prothrombin complex concentrate is most appropriate for reversing the effects of vitamin K antagonism. The evidence for the use of blood components to reverse the anticoagulant effects of direct oral anticoagulants (DOACs) is still poor (26). Coagulopathy in liver disease is complex, traditionally viewed as a disorder of impaired hemostasis, but it is probably more accurate to consider the coagulopathy of liver disease as a “rebalanced hemostasis,” as it can be associated with thrombotic as well as hemorrhagic outcomes (27–29, 31). There is limited evidence to suggest that raising the INR in these patients is associated with an increased bleeding risk, and limited evidence to suggest that treatment with FFP either improves coagulation parameters or reduces bleeding risk *in vivo* (29). In addition to traditional coagulation tests, some studies suggest the use of point-of-care viscoelastic tests (e.g., thrombolastography, thromboelastometry, and sonoclot) to diagnose coagulation problems in patients with liver disease (30). Point-of-care viscoelasticity tests can provide actionable targets for correcting coagulation defects in patients with bleeding liver disease and may provide evidence-based algorithms for liver disease, but it is uncertain whether it can guide the management of patients with UGIB with liver disease (30). In our study, we did not perform viscoelastic testing on enrolled patients and therefore cannot provide evidence whether viscoelastic testing analysis affects transfusion requirements and alters clinical outcomes in this high-risk patient population.

Some limitations and strengths should be considered. First, to the best of our knowledge, this is a real-world prospective cohort study based on large-scale with a tremendous sample size. Second, we used comprehensive statistical analysis, including multiple logistic regression and subgroup analysis, to detect the association between FFP transfusion and mortality or rebleeding, which make our results become reliable. However, all clinical management decisions are still made by the attending physicians at the point of care. Although the participating hospitals in this study adopted standard clinical management protocols, some treatment options showed difference between the cases. Additionally, we only collected the results of whether or not patients died within 90 days and did not collect the specific time of death of each deceased patient after admission. If time was included for analysis, it may have more important clinical value. Finally, more prospective RCTs are now required to examine these findings and establish a possible causal link between FFP transfusion and adverse outcomes in patients with UGIB.

## Conclusion

Blood is a scarce and expensive resource and there persist surprisingly large gaps in the evidence base to guide the effective use of FFP transfusion in patients with UGIB. This study suggests a clinically important association between FFP transfusion and an elevated risk of rebleeding and mortality, particularly in patients with an INR ≤1.5. FFP transfusion in acute UGIB does not improve the poor outcomes. Clinicians and expert consensus groups need to be aware of such considerations, as basic aspects of resuscitation are now being questioned and reassessed in many specialties across the world.

## Data Availability Statement

The raw data supporting the conclusions of this article will be made available by the authors, without undue reservation.

## Ethics Statement

The studies involving human participants were reviewed and approved by Ethics Committee of Peking Union Medical College Hospital. The patients/participants provided their written informed consent to participate in this study.

## Author Contributions

SL and XY: conception, design, and manuscript preparation and editing. SL: definition of intellectual content and final approval of the article. SL, XZ, and JW: literature search, analysis, and interpretation of the data. XZ: drafting of the article. XY and HZ: critical revision of the article for important intellectual content. All authors have read and approved the manuscript. All authors contributed to the article and approved the submitted version.

## Funding

The AUGUR study was initiated by the Emergency Department of Peking Union Medical College Hospital, assisted by the Chinese College of Emergency Physicians and the Chinese Emergency Medical Partnership. The authors declare that this study received funding from AstraZeneca China. The funder was not involved in the study design, collection, analysis, interpretation of data, the writing of this article or the decision to submit it for publication.

## Conflict of Interest

The authors declare that the research was conducted in the absence of any commercial or financial relationships that could be construed as a potential conflict of interest.

## Publisher's Note

All claims expressed in this article are solely those of the authors and do not necessarily represent those of their affiliated organizations, or those of the publisher, the editors and the reviewers. Any product that may be evaluated in this article, or claim that may be made by its manufacturer, is not guaranteed or endorsed by the publisher.
